# Morfo-anatomical insights into the germination and protocorm growth of the endangered *Vanilla lindmaniana* (Orchidaceae)

**DOI:** 10.1007/s10529-026-03752-2

**Published:** 2026-06-22

**Authors:** Jenifer Caroline Moreira Campos, Clarissa Alves Stefanello, Vitória Weiss Pereira Moraes, Emerson Ricardo Pansarin, Hugo Pacheco de Freitas Fraga

**Affiliations:** 1 Plant Micropropagation Laboratory, Department of Botany, Federal University of Paraná, Curitiba, Paraná Brazil; 2 Graduation Program in Botany, Federal University of Paraná, Curitiba, Paraná Brazil; 3 FFCLRP, Department of Biology, University of São Paulo, Ribeirão Preto, São Paulo Brazil

**Keywords:** BM1 culture medium, Conservation, Mature seeds, Micropropagation, Vanilloideae

## Abstract

Despite their global importance, knowledge of *Vanilla* plant nutrition and physiology remains limited, particularly regarding germination processes that underpin species ecology, fruit attraction, and seed dispersal. This study elucidates the germination stages of the endangered *Vanilla lindmaniana* through detailed morpho-anatomical analyses. Mature seeds were disinfected, chemically scarified, and sown on BM1 medium under controlled conditions (30 °C, darkness). Protocorm development was characterized through stages at morphological and anatomical levels, utilizing light microscopy and scanning electron microscopy (SEM). Successful in vitro germination was achieved, with initiation at three months and a germination rate of 61.8%, alongside a protocorm rooting frequency of 93.86%. Germination was triggered by rupture of a specific seed coat layer, resulting in a “clam-like” split, concurrent with starch accumulation. Roots emerged at Stage IV, growing vigorously, while the shoot apex remained short; raphides were occasionally observed in the root cortex. This work presents a novel inoculation and germination procedure for mature *V. lindmaniana* seeds, highlighting BM1 medium as a suitable formulation for germination and early development. Understanding these mechanisms is vital for conservation efforts and genetic engineering, facilitating species propagation and advancing knowledge of the *Vanilla* life cycle. Our findings contribute essential insights into the early development stages of this rare species, supporting both its preservation and sustainable cultivation.

**Supplementary Information:**

The online version contains supplementary material available at https://doi.org/10.1007/s10529-026-03752-2.

## Introduction

*Vanilla* Mill. (Orchidaceae) is among the most valued genera in the family, renowned not only for its ecological significance but also for producing edible fruits. The global market for vanilla reaches billions of dollars annually, as the compound is used extensively in gastronomy, perfumery, and the pharmaceutical industry (Silva-Oliveira et al. 2022; Franco et al. 2024), and is estimated to surpass USD 5 billion by 2030, driven by increasing consumer preference for natural and organic products (Grand View Research 2025). While the Mexican *Vanilla planifolia* Andrews remains the most extensively cultivated species, many Brazilian vanilla species are gaining interest due to their unique aromatic profiles (Pérez-Silva et al. 2021; Silva-Oliveira et al. 2022). Nonetheless, vanilla orchids are integral to ecosystems through complex interactions with pollinators, seed dispersers, fungi, and host plants, emphasizing the importance of foundational biological knowledge, including seed germination and development.

*Vanilla lindmaniana* Kraenzl. (*Vanilla* sect. *Xanata*) is an epiphyte orchid with long, scandent stems, native to the open forests of Amazon, Cerrado, and Pantanal biomes (Pansarin 2025c). It has recently been reappraised as an independent species from *V. palmarum* (Salzm. ex Lindl.) Lindl., with which it shares some ecological characteristics, such as the yellow flowers and phorophyte association (Pansarin 2025c). The species is found growing on the trunks and leaves of palm trees of the genera *Acrocomia* Mart., *Attalea* Kunth, and *Mauritia* L.f., but displaying preference for one or other phorophyte, depending on the environment (Barberena et al. 2019; Pansarin 2025c). While a strongly ornamental plant, *V. lindmaniana* fruits are odourless, producing fatty acids instead of aromatic compounds (Pérez-Silva et al. 2021; Pansarin 2025a).

Interest in vanilla plants has grown extensively in the scientific community in the past few years. However, topics such as “biodiversity conservation” and “cell biology” are yet the least approached, and the majority of the studies with Brazillian endemic vanilla deal with plant descriptions, as evaluated in the recent scientometric study by Nadal et al. (2025). Extinction of plants has happened in the last century at a higher rate than the natural turnover of species, sometimes even before the species are formally described and studied (Humphreys et al. 2019). Due to intense deforestation of the Brazilian Cerrado and Amazon Rainforest for the past few decades, *V. lindmaniana* was evaluated within IUCN criteria as Endangered (Pansarin 2025c), being one of the very few species whose conservation status has been accessed. Threatened taxa can only be efficiently protected if the basic knowledge regarding the plant’s life cycle is assessed, and although great advances have been made in the species’ regard (Pansarin 2025a, 2025c), there are still aspects of the plant’s biology to uncover. Germination and establishment of young plants secure the species’ survival and allow for appropriate production of in vitro plants while preserving the genetic variability of the species.

It is considered in the literature that the in vitro germination of mature seeds of *Vanilla* is difficult, because, even though the plants are epiphytes (or hemi-epiphytes), their germination patterns are reminiscent of those of terrestrial orchids (Yeh et al. 2021). Terrestrial orchids usually present dormancy, which severely reduces germination rates both in vitro and in nature, often referred to as a morpho-physiological dormancy due to a combination of factors regarding the accumulation of inhibitory substances and integument waterproofing (Swarts and Dixon 2009; Magrini and De Vitis 2017). In *Vanilla*, seed dormancy is generally attributed to the thick outer seed coat built of phytomelanin, a catechyl-lignin type that darkens the seed coat very early at fruit development, frequently embedded in phenolic compounds (Chen et al. 2012; Yeung 2017; Yeh et al. 2021). This hard seed coat may restrain germination through impaired gas exchange and water impermeability (Meyer and Witmer 1998; Pansarin 2021).

Recent evidence indicates that *Vanilla* seeds are dispersed by animals (Pansarin 2021, 2024, 2025a, 2025b; Pansarin and Suegutsu 2022; Karremans et al. 2023a, 2023b). In fact, chemical scarification was shown to be required to break seed dormancy, enhancing germination and synchronizing the biological process involved in the germination (Pansarin 2021; Pansarin and Suegutsu 2022). Indeed, hard-coated seeds are typically adapted to survive gut passage (Kleyheeg et al. 2018). Germination of mature *Vanilla* seeds has been achieved through various chemical scarification methods, including hydrochloric acid (Šoch et al. 2023), sodium hypochlorite (Yeh et al. 2021; Chaipanich et al. 2020), and sulfuric acid (Pansarin 2021). However, seeds of *V. lindmaniana* recovered from bird faeces showed similar germination rates to non-scarified seeds (Pansarin 2025a), and *V. planifolia* seeds exhibited equally low germination regardless of treatment (Karremans et al. 2023a). Notably, digested seeds germinated earlier than undigested ones (Pansarin 2025a), suggesting that *V. lindmaniana* germination may depend on factors beyond mere scarification.

Building on these insights, this study aims to: (i) establish an efficient protocol for seed surface sterilization and in vitro germination of *V. lindmaniana*; (ii) elucidate the in vitro germination mechanism of an epiphyte *Vanilla* species; (iii) characterize and compare seed micromorphology within the genus; and (iv) analyse protocorm development at morphological and anatomical levels. These objectives seek to advance understanding of *V. lindmaniana* early life stages, supporting conservation and propagation efforts.

## Materials and methods

### Plant material

Fully mature fruits (dark pods split open, drying at the stem) of *Vanilla lindmaniana* were harvested from adult plants grown at the LBMBP Orchid House (Orquidário do Laboratório de Biologia Molecular e Biossistemática de Plantas), University of São Paulo, Ribeirão Preto municipality, Brazil. The plants were subjected to manual self-pollination and monitored for capsule development. A voucher specimen of *V. lindmaniana* was deposited in the dried collection of the LBMBP Orchidarium, under registry number VAN034. The pods were stored at room temperature (± 25 °C) and kept in dark and dry conditions for about six months until ready to use.

### Disinfection and germination procedure of seeds

Disinfection procedure was adapted from Šoch et al. (2023). The mature seeds were scooped out of the dry, open capsules, placed in aluminium envelopes, and immediately carried to a laminar airflow hood to begin disinfection process. Then, they were transferred to autoclaved white polyester voile pieces, which were tied into small bags with autoclaved white cotton thread. Each bag contained around 100 mg of seeds (hereinafter referred to as ‘seed bag’). The seed bags were immersed in ethanol 70%, 15 min, washed with autoclaved water, then immersed in HCl 0.1 M, 1 h, washed again, and finally immersed in NaOCl 2% + Tween20 0.1%, with a final wash in autoclaved water. The seed bags were cut open with sterile scissors, and the seeds (around 50 mg, ± 1200 seeds) were inoculated in Petri dishes containing 25 mL of BM1 (Van Waes & Debergh 1986) culture medium gelled with 7 g.L^−1^ agar (IONLAB, Brazil) with the aid of a sterile scalpel and spatula (Figure S1). The culture medium was previously adjusted to pH 5.8 and autoclaved at 121 °C for 20 min. The Petri dishes were kept in complete darkness and controlled temperature of 30 ± 1 °C. Protocorm emergence was observed monthly, and germination rates were calculated as the number of germinated seeds/total number of seeds * 100. Germination stages were recorded according to Alomía et al. (2017), with modifications. Data was calculated as the average of six repetitions.

### Light microscopy

Seed and protocorm samples were immersed in a fixation solution of 2.5% paraformaldehyde and 0.1 M phosphate buffer and stored in the cold (4 ± 1 °C) for 24 h. Then, the samples were twice washed with phosphate buffer and dehydrated in ethanol series (30°, 50°, 70°, 90°, 100°), twice for 15 min at each step, the last being twice for 30 min. Then, the samples were infiltrated with Historesin® (Leica Biosystems, Germany) at an increasing ratio of Historesin®: ethanol in the proportions of 1:2, 1:1 and 2:1, for two hours each, before final infiltration with pure Historesin® in a vacuum oven for 48 h. Finally, the samples were embedded in Historesin® and the manufacturer’s hardener (15:1) and kept in an oven at 40 °C until completely dry. The material was submitted to sectioning in an Olympus CUT 4055 rotation microtome (Olympus America Inc., USA) in 10 μm sections. For histochemical tests, slides were treated with 0.05% Toluidine Blue O in phosphate buffer solution pH 6.8 (O’Brien et al. 1964); Ferric Chloride 10% (Johansen 1940); Lugol solution (Kraus et al. 1998); and Phloroglucinol-HCl (Herr 1992), finished with synthetic Canadian Balm (CRQ Química, Brazil). Micrographs were obtained with the Olympus DP72 digital camera coupled to the Olympus BX50 optical microscope at the Centro de Tecnologias Avançadas em Fluorescência (CTAF), Federal University of Paraná.

### Scanning electron microscopy

Seed and protocorm samples were immersed in a fixation solution of 2.5% paraformaldehyde and 0.1 M phosphate buffer and stored in the cold (4 ± 1 °C) for 24 h. Then, the samples were twice washed with phosphate buffer and dehydrated in an ethanol series as described previously. The samples were subjected to CO_2_ critical point drying (BAL-TEC CPD 030, Columbia University, USA) and covered with gold dust before analysis in a MIRA3 TESCAN Scanning Electron Microscope (TESCAN, Czech Republic). Analysis and photography were carried out at the Centro de Microscopia Eletrônica (CME), UFPR.

## Results

**Fig. 1:**
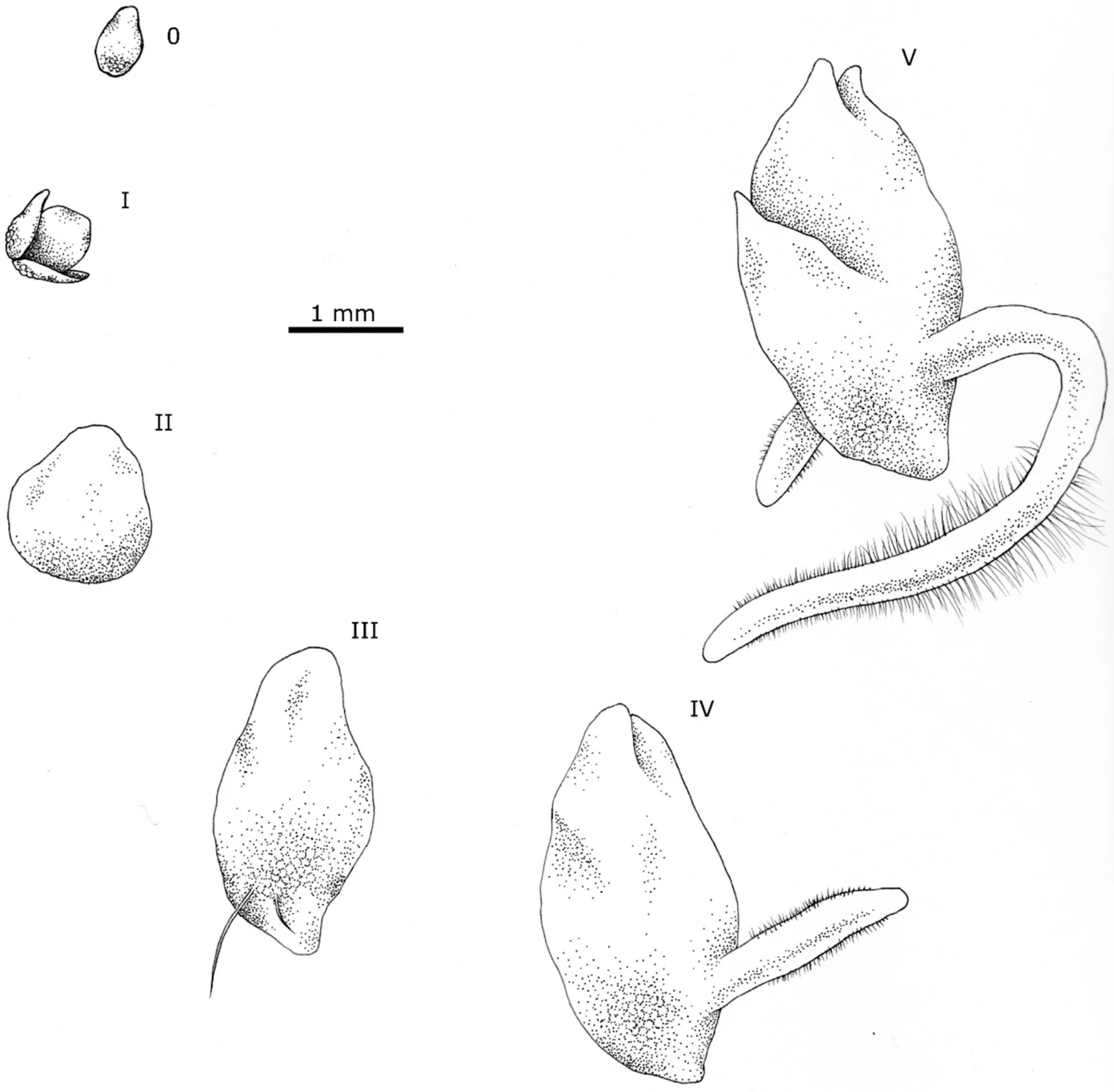
Seed germination and protocorm developmental growth stages (**0**–**V**) of *Vanilla lindmaniana*. **0**: dark seed, not germinated. **I**: rupture of the seed coat (= germination). **II**: enlarging embryo with evident protomeristem. **III**: protocorm increase (ten times), occasional rhizoid emergence. **IV**: emergence of the first root. **V**: root with more than 5 mm in length. Scale bar = 1 mm

The surface sterilization procedure as described proved to be the most efficient, as we achieved no contamination and nearly zero seed losses during handling of the material. *Vanilla lindmaniana* seeds began to germinate three months after inoculation, and new protocorms continued to emerge in the following months. The majority of protocorms emerged at the third and fourth months of cultivation. Thus, seven months after sowing, germination rates reached 61.8%. Five growth stages (Fig. 1) were identified during protocorm development. Rhizoid emergence began late, at Stage III, but many protocorms did not present rhizoids at all. Regardless, nearly all of them (93.86%) developed roots. Protocorms grew rapidly after the embryo had ruptured the seed coat, growing from Stage I to Stage IV in less than a month.

**Fig. 2:**
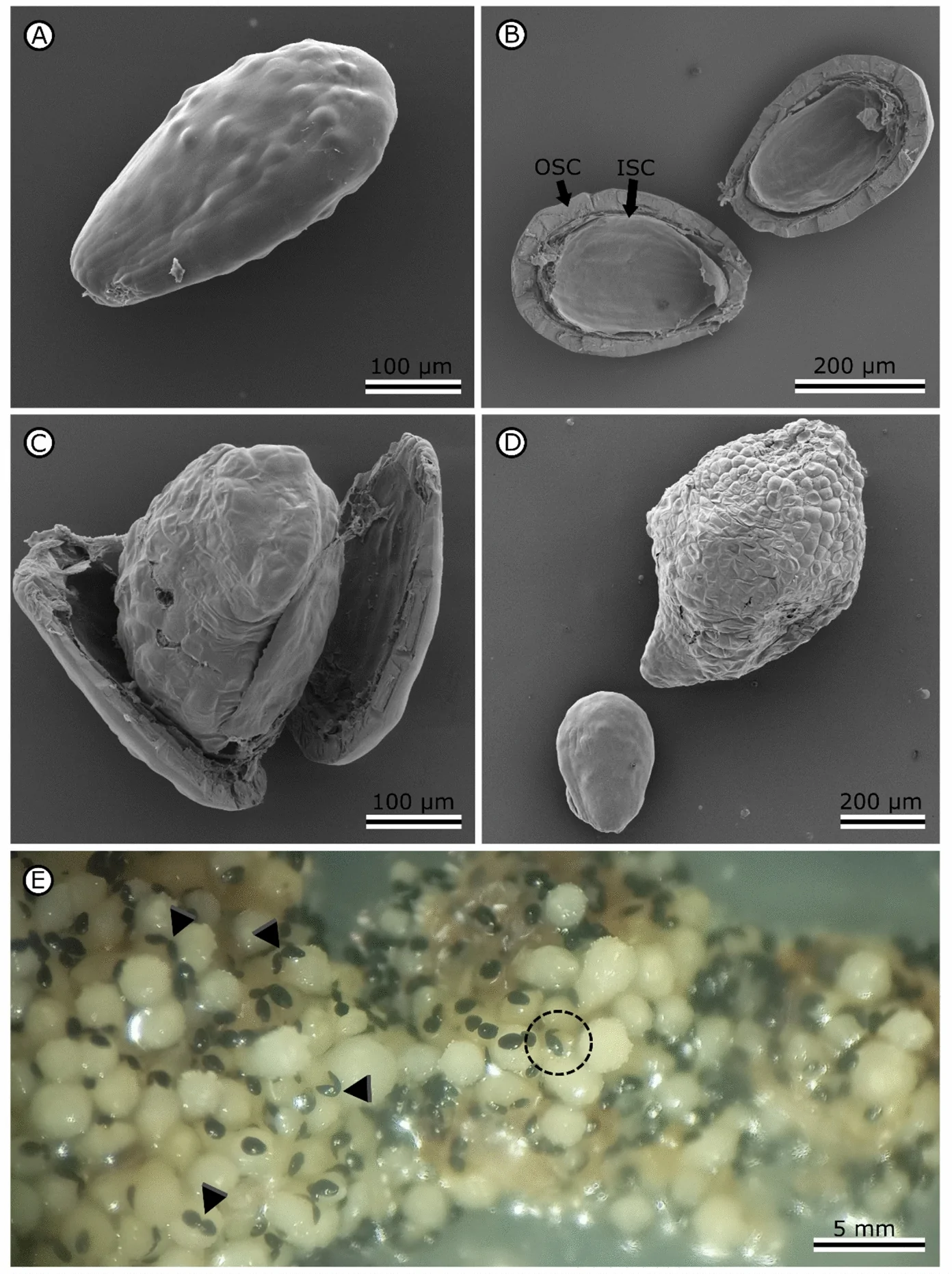
Germination process of *Vanilla lindmaniana*, Scanning Electron Microscopy (**A**–**D**) and protocorm overview (**E**). **A**: *V. lindmaniana* seed at maturity. **B**: Seed split in half, showing the internal layers. **C**: Seed rupture during germination, notice the protocorm at Stage I. **D**: Size comparison between the seed and a protocorm at Stage II. **E**: Stereomicroscope view of the germination at three months after seed sowing. The circle shows a protocorm at Stage I. Arrowheads point to seeds split in the ‘clam-like’ shape, halves still attached at the chalazal end. *ISC* inner seed coat, *OSC* outer seed coat

**Fig. 3:**
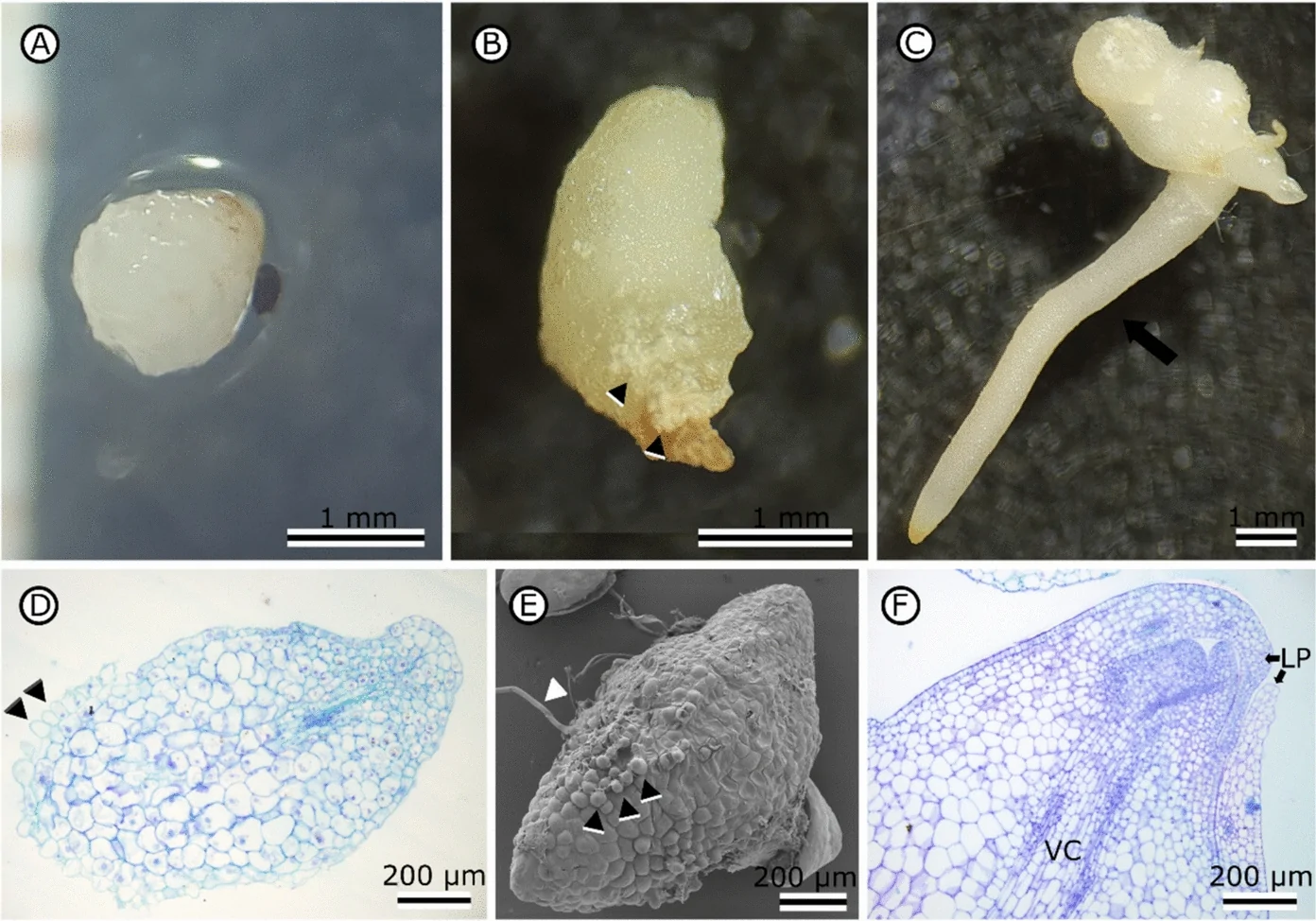
Protocorm growth of *V. lindmaniana*. **A**: Protocorm at Stage II. **B**: Protocorm at Stage III. Black arrowheads point to the cells in the lower portion, which have the potential of forming rhizoids. **C**: Protocorm at Stage IV. The arrow points to the first true root. **D**: Longitudinal section of a Stage III protocorm, stained with Toluidine Blue O dye. Black arrowheads point to the cells in the lower portion, which have the potential of forming rhizoids. **E**: Scanning Electron Microscopy of a Stage III protocorm. Black arrowheads point to the same cells as seen in **D**. The white arrowhead points to a rhizoid. **F**: Longitudinal section of the shoot tip of a Stage IV protocorm, stained with Toluidine Blue O dye. *LP* leaf primordia, *VC* vascular cylinder

Seeds of *V. lindmaniana* are ovoid in shape; at germination, the seeds open in two symmetrical halves alongside de longitudinal axis, starting from the micropyle where the seed connects with the placenta, in a “clam-like” split (Fig. 2A–C, E arrowheads). The breakage line is evident even in intact seeds, though cell borders are not clear. On the outside, the seed presents protuberances at the lower thirds (Fig. 2A). Microscopy analyses revealed the presence of a thin inner seed coat immediately surrounding the embryo and an outer seed coat, thickened and hardened, enveloping the whole seed except at the micropyle (Fig. 2B, C). The outer and inner seed coats measure 30.6 and 12.05 μm on average, respectively. Rapid growth takes place once the protocorm has emerged from the seed coat, with a ten-fold increase in size up to Stage III (Figs. 2C, D, 3A–C). Starch granules were seen all over protocorm cells and the root cortex. Protocorms at Stage III have several cells at the lower portion with the potential of forming rhizoids (Fig. 3D, E), even if the actual development of this tissue does not take place. Instead, a single true root emerges and elongates vigorously, often up to 10 cm before a second root is initiated. Root hairs cover the entire length of the roots, and raphides were occasionally seen in cortex cells. Velamen was absent during the time of the experiment evaluation. The apex itself remains very short (> 5 mm) for several months but rapidly becomes chlorophyllate when exposed to white light. Transition from darkness to light did not affect root growth in any sense. Histochemical tests for phenolic compounds and lignin were negative at all Stages.

## Discussion

Previously, in a pilot experiment with a similar species (*Vanilla phaeantha*), we tested four different time exposures (30 min, 1 h, 2 h and 4 h) to HCl, following the experimental guidelines from Šoch et al. (2023) with *V. planifolia*, and an additional treatment of soaking in pure water for 4 h. Few protocorms germinated in all treatments (data not shown). SEM analysis did not reveal any scratches or damage caused to the seed coat regardless of the time exposure or reagent (Figure S2), which agrees with the observations by Šoch et al. (2023), who reported no significant differences in seed germination from the exposure of 30 min or 4 h to HCl. Thus, we chose the 1 h soaking time (HCl) to ensure better disinfection. Moreover, having HCl as the last reagent during seed disinfection resulted in utmost failure; hence, the steps were inverted to the ethanol: HCl: NaOCl sequence as described.

The structure of *V. lindmaniana* seeds is very similar to other *Vanilla* species. The seeds share the occurrence of protuberances on the outer seed coat with *V. imperialis* Kraenzl. (Gamarra et al. 2018) and *V. oroana* Dodson (Alomía et al. 2016), but not with *V. planifolia* (Yeh et al. 2021), nor *V. calyculata* Schltr., *V. odorata* C. Presl or *V. rivasii* Molineros, R.T. González, Flanagan & J.T. Otero (Alomía et al. 2016), all of which have a smooth testa. *Vanilla lindmaniana* emerges in a basal position as sister to the remaining *Vanilla* with non-membranaceous leaves, while the *Vanilla* species with smooth seed coats emerges in a most derivate position in such clade (Pansarin 2025d). The protuberances may aid in maintaining moisture around the seed by trapping water droplets in between, thus minimizing embryo mortality by desiccation. Seed micromorphology is important to support taxonomic treatments and add to the overview of a group’s evolution and ecology (Gamarra et al. 2018). Besides, it allows more accurate identification of loose seeds in the field, which may be recovered from animal faeces and thus associated with dispersal (Alomía et al. 2016).

The ovoid shape and the clear distinction between the inner and outer seed coats are very similar to those described for *V. planifolia* (Yeh et al. 2021). Endosperm is apparently absent, which is the most common condition in orchids (Yeung 2017; Yeh et al. 2021). Starch accumulation at early growth stages has been observed in some species (Lee et al. 2013; Ferreira et al. 2018) and may be a strategy that compensates for the lack of endosperm to ensure protocorm growth.

The clam-like split the seeds undergo for germination implies that the breakage line represents a thinner, more fragile section of the seed coat, which, under intense water or gas pressure, should crack open to release the enlarging protocorm. Meyer and Witmer (1998) also observed such a condition from the hard seeds of *Viburnum dentatum* L. (Viburnaceae) and *Prunus virginiana* L. (Rosaceae), both of which are consumed by birds. The authors also mention that scarification of the endocarp within the birds’ guts did not improve germination of those species and point out that a variety of mechanisms balance seed dormancy, and that local environment quality may be as influential (Meyer and Witmer 1998). Abiotic factors such as temperature and substrate moisture can break seed dormancy, especially for those that are dependent on a particular season and have a shorter time frame for successful germination and seedling recruitment (Rasmussen et al. 2015). Chemical and/or physical scarification of the seeds should be considered carefully, because damage to areas outside of the breakage line may not aid much in germination and will possibly hurt the embryo. Seeds ingested by birds must survive a tough mechanical (by the gizzard) and chemical (by the gut) digestion, and are often too damaged to germinate (Kleyheeg et al. 2018). *Vanilla* seeds, being very tiny, may avoid most of the mechanical scarification by dodging the gizzard movements, hence facing mainly the acid digestion, which is in accordance with evidence that the smallest seeds have the highest potential to be efficiently dispersed by birds (Soons et al. 2008; Kleyheeg et al. 2018). The disinfection procedure shown in this work was safe enough not to impair the germination of *V. lindmaniana.* Moreover, the use of chemical agents is helpful to clean the seeds from the fruit pulp, which then improves water uptake and gas exchange between the seeds and the medium, thus enhancing germination (Meyer and Witmer 1998).

Pansarin (2025a) reported that in vitro germination of *V. lindmaniana* mature seeds was achieved within five to seven months, for the seeds that had been expelled in birds’ faeces, and a rate of ± 32% over a year. In the present work, germination began three months after sowing and reached ± 62% in seven months. In parallel, Šoch et al. (2023) obtained germination of *V. planifolia* (± 60%), but only evaluated it at five months of cultivation, making it unclear when germination had started. As chemical scarification was performed on all mentioned experiments (either by the birds or with hydrochloric acid), it is possible to assume that the environmental aspects of the cultivation (culture medium composition, temperature, and light exposure) had a greater effect upon seed dormancy.

In the previous work with *V. lindmaniana*, half-strength MS medium (Murashige and Skoog 1962) was used, and seeds were placed in a growth chamber at 25 ± 2 °C and a photoperiod of 16 h (Pansarin 2025a). Yeh et al. (2021) also used those growth conditions with *V. planifolia,* recovering ± 10% of protocorms. In the current work, BM1 (Van Waes and Debergh 1986) culture medium was chosen, and the seeds were cultivated in a 30 ± 1 °C chamber in complete darkness, following Šoch et al. (2023). Cultivation on BM1 medium ensured more than 60% of germinated protocorms for both *V. lindmaniana* and *V. planifolia*. Although BM1 was originally developed for terrestrial orchids (Van Waes and Debergh 1986), epiphytes may also benefit from its lower salts and exclusively organic nitrogen compounds (such as casein, glutamine, and glycine), as protocorm establishment is fundamentally promoted by organic nitrogen (Arcidiacono et al. 2021). Moreover, *Vanilla* seeds have a similar germination pattern to terrestrial orchids (Yeh et al. 2021). In their natural habitats, epiphytes are adapted to fast-shifting environments and low mineral availability, which allows them to thrive in poorer conditions. At the same time, this adaptation may turn young seedlings fairly sensitive to high salt conditions, to the point of growth inhibition. Many authors have achieved higher germination rates for both terrestrial and epiphytic orchids in lower salt media (Van Waes and Debergh 1986; Santos et al. 2016; Zeng et al. 2015; Magrini and De Vitis 2017; Melo Ferreira et al. 2017; Vudala and Ribas 2017; Diantina et al. 2020; Koene et al. 2020; Arcidiacono et al. 2021). Hence, the high germination rate obtained for *V. lindmaniana* shows that this species also requires fewer nutrients during protocorm establishment.

Most growth chambers for in vitro plants have a temperature of around 23–25 °C. Šoch et al. (2023) proposed the innovation of germinating *Vanilla* seeds at 30 °C, as the authors had not obtained any germination at the usual 25 °C. Based on the fact that *V. lindmaniana* is a tropical plant whose fruit matures by the end of the winter (Pansarin 2025c), when temperatures above 30 °C become increasingly frequent in the Amazon and Cerrado biomes, it was reasonable that the species would benefit from such conditions to germinate. Thus, it is reinforced that the species’ habitat and lifestyle are of utmost importance for the development of appropriate culture conditions in vitro.

## Conclusion

A high germination rate of *Vanilla lindmaniana* mature seeds was achieved on BM1 culture medium and complete darkness. The germination mechanism suggests the seeds undergo intense pressure before splitting and releasing the protocorm, which is likely related to water uptake. The seed micromorphology and protocorm characterization shown in this work should aid in the recognition of the species in field work and further research regarding its germination patterns. Environmental aspects regarding temperature, humidity, and nutrient availability play a major role in *V. lindmaniana* germination. As acid scarification of the seed coat is not strictly required for germination, research should be aimed at investigating other forms of dormancy, either related to hormonal balance, temperature cycling, and/or water availability. Other media formulation, drying agents and plant growth regulators could further clarify the species’ requirements and aid in the comprehension of the vanilla plants’ cultivation.

## Supplementary Information

Below is the link to the electronic supplementary material.Supplementary file1 (PNG 9858 KB)Supplementary file2 (PNG 5869 KB)Supplementary file3 (DOCX 3467 KB)

## Data Availability

No datasets were generated or analysed during the current study.
