# Supplementary figures and images for: Morfo-anatomical insights into the germination and protocorm growth of the endangered Vanilla lindmaniana (Orchidaceae)
Source: Biotechnol Lett. 2026 Jun 22;48(4):81. doi: 10.1007/s10529-026-03752-2 (PMC13287153; doi:10.1007/s10529-026-03752-2)

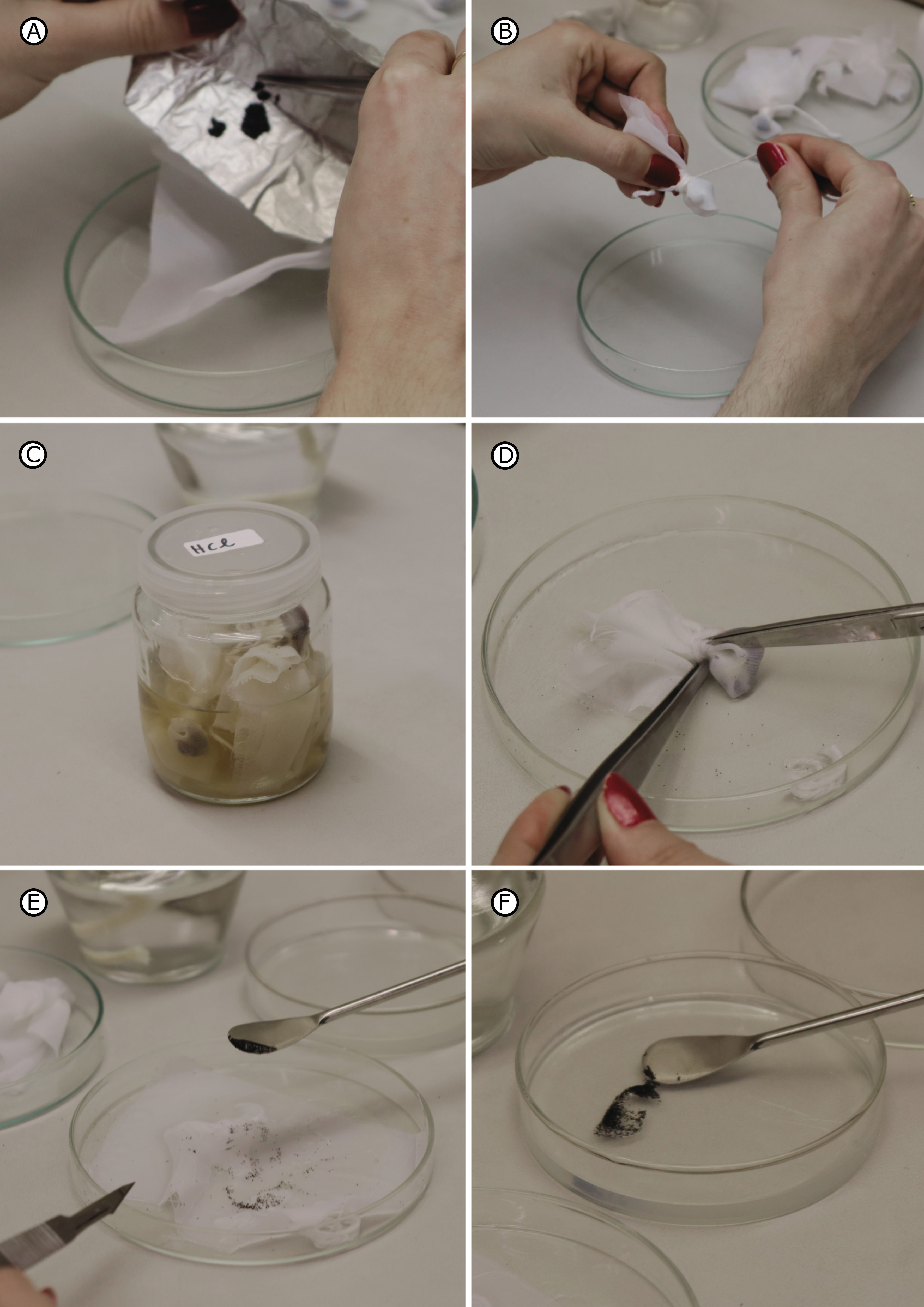

Supplement: Supplementary file 1 — Supplementary file1 (PNG 9858 KB) [file 10529_2026_3752_MOESM1_ESM.png]

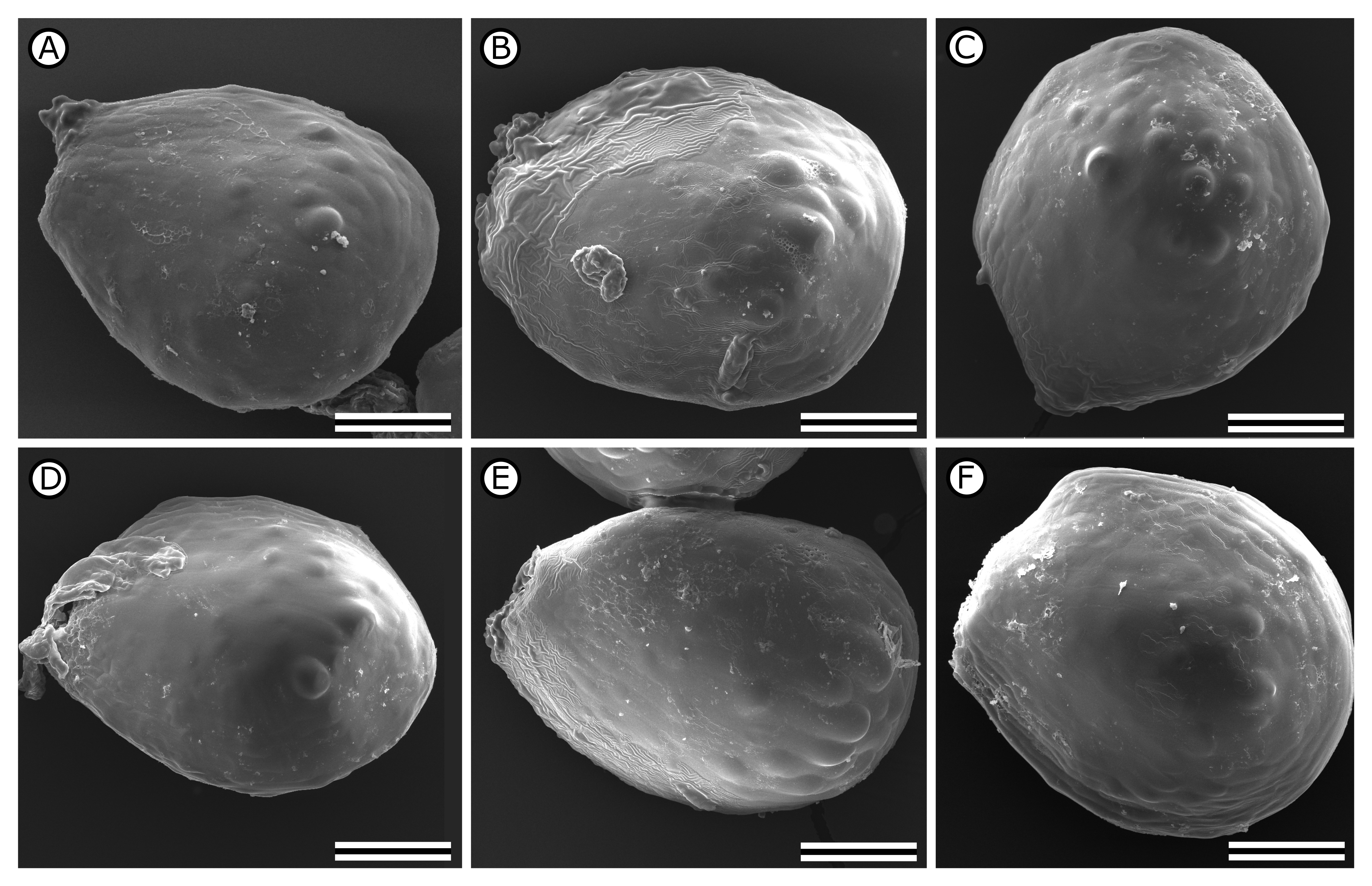

Supplement: Supplementary file 2 — Supplementary file2 (PNG 5869 KB) [file 10529_2026_3752_MOESM2_ESM.png]
