# Supplementary material for: Morfo-anatomical insights into the germination and protocorm growth of the endangered Vanilla lindmaniana (Orchidaceae)
Source: Biotechnol Lett. 2026 Jun 22;48(4):81. doi: 10.1007/s10529-026-03752-2 (PMC13287153; doi:10.1007/s10529-026-03752-2)
Supplement: Supplementary file 3 — Supplementary file3 (DOCX 3467 KB) [file 10529_2026_3752_MOESM3_ESM.docx]

Supplementary material to: Morfo-anatomical insights into the germination and protocorm growth of the endangered *Vanilla lindmaniana* (Orchidaceae)

Clarissa Alves Stefanello^1,2^, Jenifer Caroline Moreira Campos^1^, Vitória Weiss Pereira Moraes^1^, Emerson Ricardo Pansarin^3^, Hugo Pacheco de Freitas Fraga^1,2^

^1^Plant Micropropagation Laboratory, Department of Botany, Federal University of Paraná, Curitiba, Paraná, Brazil

^2^Graduation Program in Botany, Federal University of Paraná, Curitiba, Paraná, Brazil

^3^University of São Paulo, FFCLRP, Department of Biology, Ribeirão Preto, São Paulo, Brazil

Corresponding author: [hugofraga@ufpr.br](mailto:hugofraga@ufpr.br) (Hugo Pacheco de Freitas Fraga)


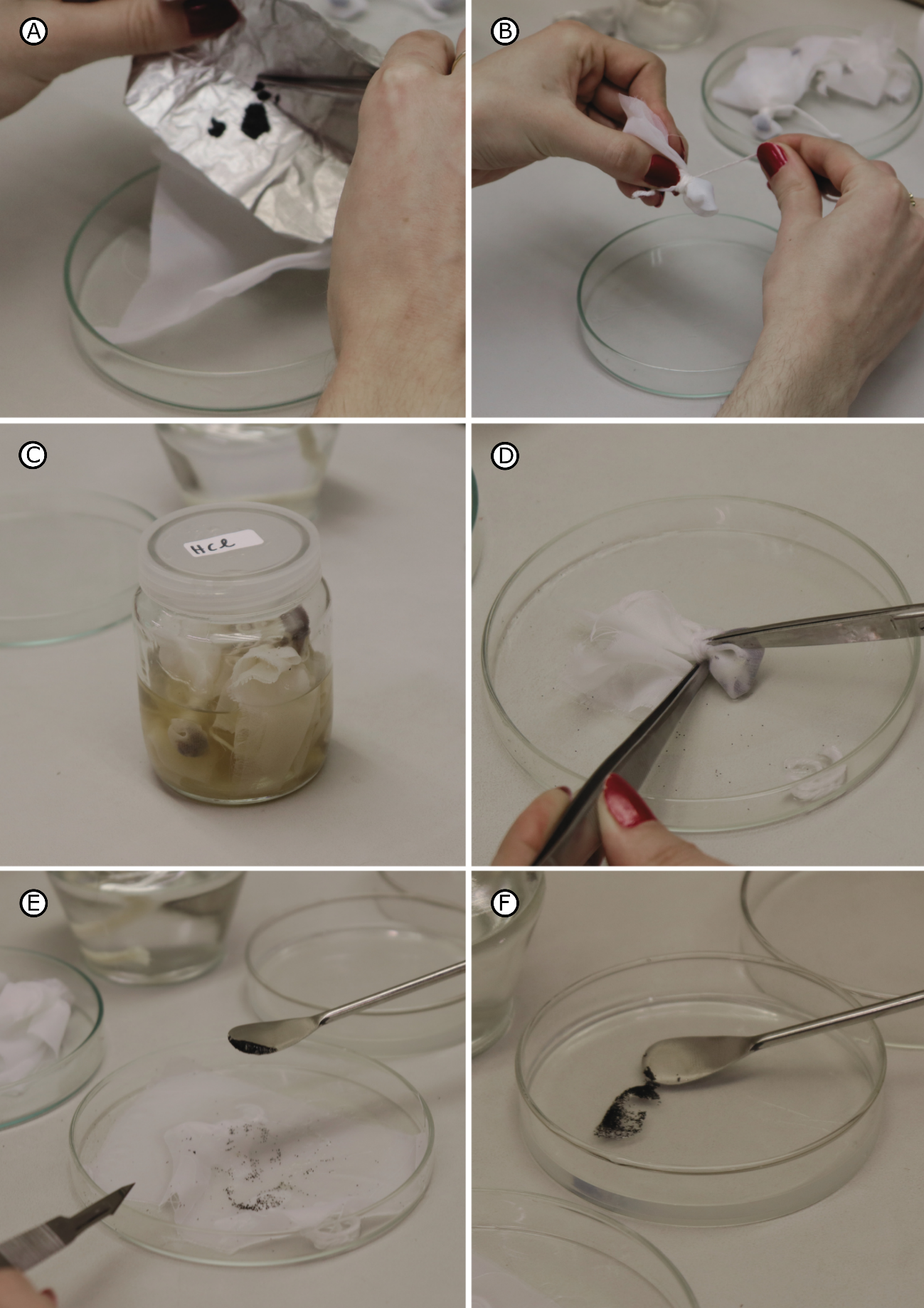


**Fig. S1** Disinfection and germination procedure of *Vanilla lindmaniana* seeds. A: After being scooped out of capsules and placed in aluminium envelopes, the seeds were taken to laminar air flow hood and transferred to autoclaved voile. B: The voile is firmly tied with autoclaved thread into the seed bag. C: Seed bags undergo immersion in different reagents (ethanol, HCl and NaOCl) and several washes in autoclaved distilled water. D: The seed bag is cut open at the thread with sterilized scissors to release the seeds. E: Seeds are removed from the bag with the aid of sterile scalpel and spatula. F: Seeds are carefully spread upon the culture medium with the sterile spatula


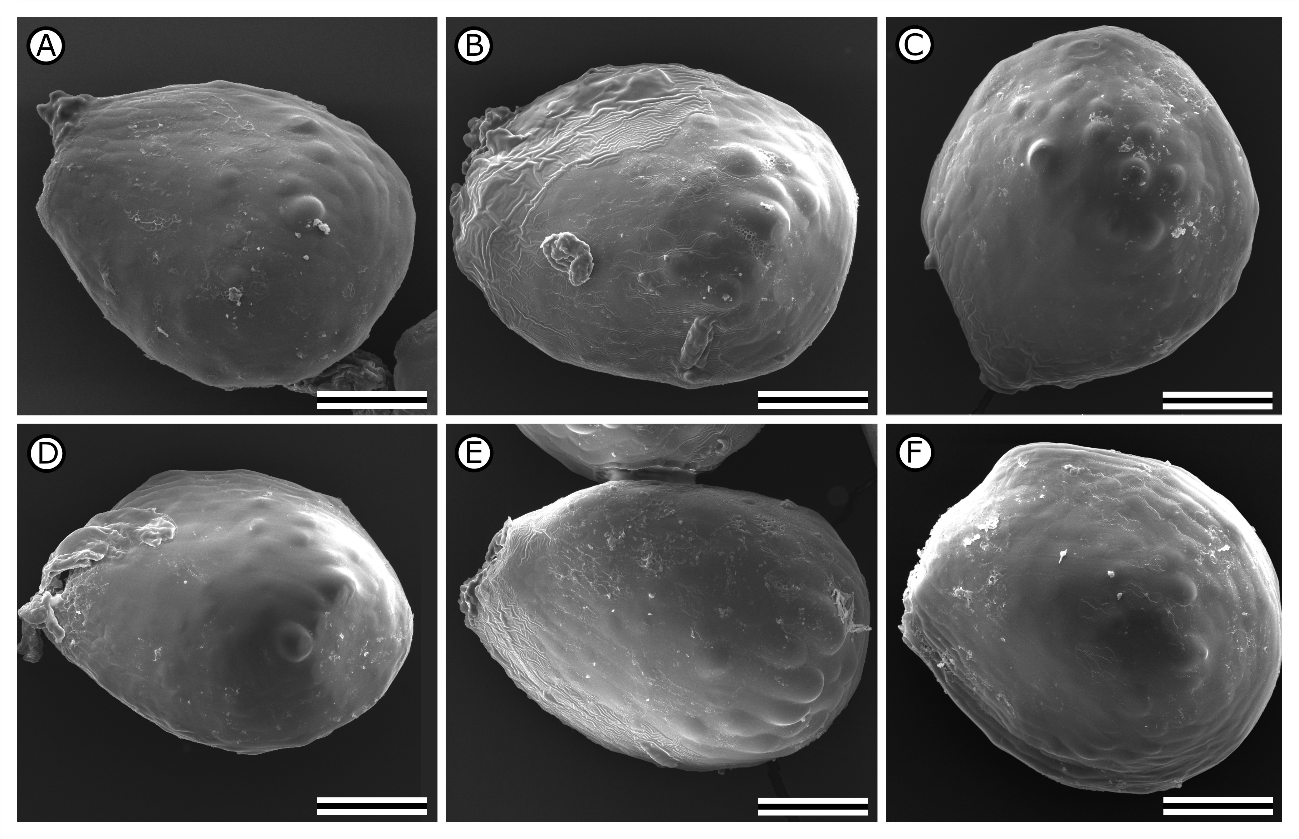


**Fig. S2** Scanning Electron Microscopy of *Vanilla phaeantha* seeds submitted to different time exposures to HCl or pure water. A: Intact seed not treated. B: 30 min HCl. C: 1 h HCl. D: 2 h HCl. E: 4 h HCl. F: 4 h pure water. Scale bar = 100 μm
